# *PIK3CA* mutation, reduced AKT serine 473 phosphorylation, and increased ERα serine 167 phosphorylation are positive prognostic indicators in postmenopausal estrogen receptor-positive early breast cancer

**DOI:** 10.18632/oncotarget.24845

**Published:** 2018-04-03

**Authors:** Naoko Ishida, Motoi Baba, Yutaka Hatanaka, Kanako Hagio, Hiromi Okada, Kanako C. Hatanaka, Kenichi Togashi, Yoshihiro Matsuno, Hiroko Yamashita

**Affiliations:** ^1^ Department of Breast Surgery, Hokkaido University Hospital, Kita-ku, Sapporo 060-8648, Japan; ^2^ Department of Surgical Pathology, Hokkaido University Hospital, Kita-ku, Sapporo 060-8648, Japan; ^3^ Research Division of Companion Diagnostics, Hokkaido University Hospital, Kita-ku, Sapporo 060-8648, Japan; ^4^ Roche Diagnostics K.K., Konan, Minato-ku, Tokyo 108-0075, Japan

**Keywords:** breast cancer, estrogen receptor, PIK3CA, AKT Ser473, ERα Ser167

## Abstract

Although endocrine therapy is the most important treatment option in estrogen receptor (ER)-positive breast cancer, new strategies, such as molecular targeted agents together with endocrine therapy are required to improve survival. *PIK3CA* is the most frequent mutated gene in ER-positive early breast cancers, and *PIK3CA* mutation status is reported to affect activation of AKT and ERα. Moreover, recent studies demonstrate that patients had a better prognosis when tumors expressed ER, androgen receptor (AR), and vitamin D receptor (VDR). In this study, we examined expression of AR and VDR, phosphorylation of AKT serine (Ser) 473 (AKT phospho-Ser473) and ERα Ser167 (ERα phospho-Ser167) by immunohistochemistry in ER-positive, HER2-negative early breast cancer. *PIK3CA* gene mutations were also detected in genomic DNA extracted from tumor blocks. Correlations between these biological markers, clinicopathological factors and prognosis were analyzed. Levels of AKT phospho-Ser473 were significantly higher in premenopausal women than in postmenopausal women. In contrast, AR expression was significantly higher in postmenopausal women than in premenopausal women. *PIK3CA* mutations were detected in 47% in premenopausal women and 47% in postmenopausal women. Postmenopausal women with *PIK3CA* wild-type tumors had significantly worse disease-free survival than patients with *PIK3CA* mutant tumors. Low levels of AKT phospho-Ser473 and high levels of ERα phospho-Ser167 were strongly associated with increased disease-free survival in postmenopausal women. Evaluation of ERα activation, in addition to *PIK3CA* mutation status, might be helpful in identifying patients who are likely to benefit from endocrine therapy alone versus those who are not in postmenopausal ER-positive early breast cancer.

## INTRODUCTION

Endocrine therapy is the most important treatment option for women with estrogen receptor (ER)-positive breast cancer [1, 2]. Nevertheless, many early breast cancer patients with tumors expressing high levels of ER relapse after initial treatments, including adjuvant endocrine therapy. We recently identified predictors of early and late distant recurrence following an analysis of clinicopathological factors and adjuvant therapies among patients with early and late distant recurrence and patients without recurrence in ER-positive, HER2-negative breast cancer [3]. Approximately two-thirds of patients who relapsed within 5 years had received anthracyclins and/or taxanes as adjuvant or neoadjuvant chemotherapy, in addition to adjuvant endocrine therapy. Thus, new strategies, such as those deploying inhibitors of oncogenic driver proteins together with endocrine therapy are required to improve survival.

Mutations of the phosphatidylinositol-4,-5-bisphosphate 3-kinase catalytic subunit alpha (*PIK3CA*) gene are one of the most frequent genetic alterations in breast cancer [4, 5]. The Cancer Genome Atlas Network reported that *PIK3CA* is the most frequent mutated gene in the luminal A (49%) and luminal B (32%) breast cancer subtypes [4]. Almost 95% of mutations occur within the helical domain (exon 9, commonly E542 and E545) and the kinase domain (exon 20, commonly H1047) [6]. A meta-analysis involving 26 studies found a significant association between *PIK3CA* mutations and expression of ER and progesterone receptor (PgR), and suggested that the clinical implications of *PIK3CA* mutations may vary according to the exons harboring the mutation [7]. However, there is currently insufficient evidence to recommend routine genotyping of *PIK3CA* in clinical practice, although many studies have been performed regarding the prognostic and therapeutic implications of *PIK3CA* mutations in breast cancer [8].

We previously showed that increased phosphorylation of ERα serine (Ser) 167 was associated with an improved survival in ER-positive early breast cancer [9]. Moreover, patients with increased ERα Ser167 phosphorylation in primary breast tumors responded to endocrine therapy, and survived significantly longer after relapse in metastatic breast cancer [10]. ERα Ser167 is phosphorylated by several kinases, including MAPK, AKT, and p90 ribosomal S6 kinase [11]. *PIK3CA* mutation status might affect phosphorylation of both AKT and ERα Ser167. In addition, our previous study demonstrated that the expression of estrogen and progesterone-responsive genes, ER-related genes and p53 in ER-positive, HER2-negative breast cancer differ between pre- and postmenopausal women. This indicates that menopausal status might affect the development and estrogen-dependent growth of ER-positive breast cancer [12].

On the other hand, Santagata and colleagues compared 3,157 human breast tumors to normal cell types and divided them into four major subtypes that were differentiated by ER, androgen receptor (AR), and vitamin D receptor (VDR) status [13]. The authors found that patients had a better prognosis when tumors expressed all three receptors. AR is detected in about 30% of triple-negative breast cancers, and its biological effects have been extensively investigated in this subtype [14]. However, although both AR and VDR are highly expressed in ER-positive breast cancer, their role in disease etiology of this subtype remains poorly understood [15–19].

In this study, we examined expression of AR and VDR, phosphorylation of AKT Ser473 (AKT phospho-Ser473) and ERα Ser167 (ERα phospho-Ser167), and the mutational status of the *PIK3CA* gene in ER-positive, HER2-negative early breast cancer tissues. We then analyzed pre- and postmenopausal women separately to determine whether there were any correlations between these biological markers, clinicopathological factors and prognosis.

## RESULTS

### Comparison of expression and phosphorylation of biological markers between pre- and postmenopausal women

**Figure 1:**
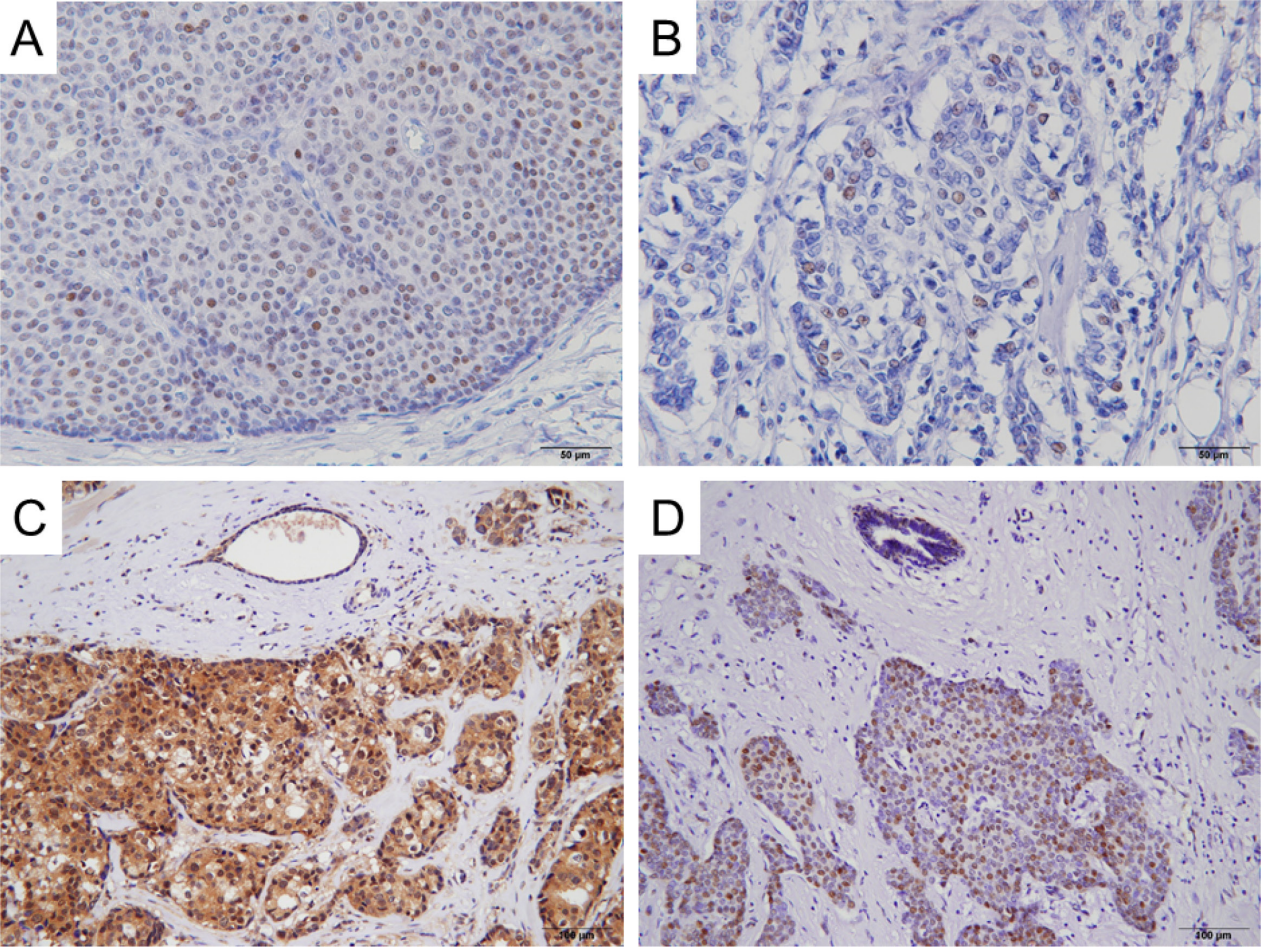
Representative immunohistochemical staining of AR, VDR, AKT Ser473 and ERα Ser167 in invasive ductal carcinoma Positive nuclear staining of AR (**A**), VDR (**B**) and ERα phospho-Ser167 (**D**), and positive cytoplasmic staining of AKT phospho-Ser473 (**C**) are seen in carcinoma cells.

We first examined the expression of Ki67, ER, progesterone receptor (PgR), AR, and VDR and the phosphorylation of AKT Ser473 and ERα Ser167 by immunohistochemistry (IHC) in breast cancer tissues in pre- (*n* = 62) and postmenopausal (*n* = 152) women (Tables 1 and 2, Figure 1). Expression of PgR and levels of AKT phospho-Ser473 were significantly higher in premenopausal women than in postmenopausal women (*P* < 0.001 and *P* = 0.014, respectively, Table 3). In contrast, the expression of AR was significantly higher in postmenopausal women than in premenopausal women (*P* < 0.001, Table 3).

**Table 1:** Clinicopathological characteristics of patients and tumors in pre- and postmenopausal women

	Premenopausal	Postmenopausal
No. of patients	62	152
Age (years), mean ± SD (range)	44.2 ± 5.5 (27–56)	63.0 ± 8.2 (42–84)
BMI, mean ± SD (range)	21.8 ± 3.7 (15.2–32.9)	24.3 ± 4.3 (14.0–40.9)
Tumor size		
T1 (≤2.0 cm)	47 (75.8%)	102 (67.1%)
T2 (2.1–5.0 cm)	13 (21.0%)	45 (29.6%)
T3 (>5.0 cm)	2 (3.2%)	5 (3.3%)
No. of positive lymph nodes		
0	42 (67.7%)	113 (74.3%)
1–3	14 (22.6%)	25 (16.4%)
≥4	4 (6.5%)	9 (5.9%)
Unknown	2 (3.2%)	5 (3.3%)
Tumor grade		
1	18 (29.0%)	32 (21.1%)
2	37 (59.7%)	107 (70.4%)
3	7 (11.3%)	13 (8.6%)
Ki67 LI		
<14%	40 (64.5%)	101 (66.4%)
14–30%	15 (24.2%)	41 (27.0%)
>30%	7 (11.3%)	10 (6.6%)
Postoperative adjuvant therapy		
None	4 (6.5%)	7 (4.6%)
Any endocrine therapy	58 (93.5%)	145 (95.4%)
Tamoxifen alone	21 (36.2%)	8 (5.5%)
Tamoxifen + LHRH agonist	25 (43.1%)	0
LHRH agonist alone	6 (10.3%)	0
Tamoxifen + LHRH agonist → AI	1 (1.7%)	0
Tamoxifen → AI	1 (1.7%)	1 (0.7%)
AI	4 (6.9%)	135 (93.1%)
AI → Tamoxifen	0	1 (0.7%)
Combined endocrine and chemotherapy	16 (25.8%)	28 (18.4%)
Follow-up (months), mean ± SD (range)	76.7 ± 38.3 (6–128)	77.7 ± 30.6 (6–129)

LI: labeling index; LHRH agonist: luteinizing hormone-releasing hormone agonist; AI: aromatase inhibitor.

**Table 2:** List of antibodies used for immunohistochemical analysis

	Antibody	Species (dilution)	2nd antibody	Evaluation
ER	SP1, Ventana Medical Systems, Tucson, USA	rabbit monoclonal (prediluted)	Ventana iVIEW DAB Detection Kit	percentage of cells showing positive nuclear staining
PgR	1E2, Ventana Medical Systems, Tucson, USA	rabbit monoclonal (prediluted)	Ventana iVIEW DAB Detection Kit	percentage of cells showing positive nuclear staining
HER2	4B5, Ventana Medical Systems, Tucson, USA	rabbit monoclonal (prediluted)	Ventana iVIEW DAB Detection Kit	0, 1+, 2+, 3+
Ki67	MIB-1, DAKO, Glostrup, Denmark	mouse monoclonal (1:200)	Dako EnVison FLEX system	labeling index
AR	AR27, Novocastra, Newcastle, UK	mouse monoclonal (1:50)	Dako EnVison FLEX system	percentage of cells showing positive nuclear staining
VDR	NBP1-19478, Novus, Littleton, USA	rabbit polyclonal (1:500)	Dako EnVison FLEX system	percentage of cells showing positive nuclear staining
AKT phospho-Ser473 (pAKT)	D9E, Cell Signaling, Beverly, USA	rabbit monoclonal (1:50)	Dako EnVison FLEX system	percentage of cells and intensity showing positive cytoplasmic staining
ERα phospho-Ser167 (pER)	GTX50140, GeneTex, Irvine, USA	rabbit polyclonal (1:50)	Dako EnVison FLEX system	percentage of cells showing positive nuclear staining

**Table 3:** Comparison of expression and phosphorylation levels of biological markers between pre- and postmenopausal women

	Premenopausal (mean ± SD)	Postmenopausal (mean ± SD)	*P*
Ki67 LI (%)	14.1 ± 13.1	11.9 ± 9.7	0.43
ER (%)	76.6 ± 25.0	81.3 ± 22.1	0.19
PgR (%)	63.5 ± 36.1	30.2 ± 32.1	<0.001^*^
AR (%)	21.9 ± 25.3	38.5 ± 31.6	<0.001^*^
VDR (%)	15.3 ± 17.7	16.8 ± 14.9	0.29
pAKT (score)	127.3 ± 86.8	94.1 ± 75.1	0.014^*^
pER (%)	24.1 ± 20.9	20.9 ± 18.7	0.27

LI: labeling index; pAKT: AKT phospho-Ser473; pER: ERα phospho-Ser167.

^*^P < 0.05 is considered significant.

### *PIK3CA* mutation frequencies in breast cancer tissues

We next evaluated the genomic DNA of primary breast cancer specimens for *PIK3CA* mutations using the cobas^®^ PIK3CA Mutation Test (Table 4). Two tumors (3.2%) in premenopausal women and 8 tumors (5.3%) in postmenopausal women were invalid. Of the 60 tumors that we were able to evaluate in premenopausal women, 31 tumors (51.7%) had no mutation (wild-type), 26 tumors (43.3%) had a single mutation, and 3 tumors (5.0%) had mutations at two sites. On the other hand, of the evaluable 144 tumors in postmenopausal women, 73 tumors (50.7%) had no mutation (wild-type), 64 tumors (44.4%) had a single mutation, 6 tumors (4.2%) had mutations at two sites, and one tumor (0.7%) had mutations at three sites. Therefore, the frequencies of single and double mutations were similar between pre- and postmenopausal women. The H1047 missense mutation was the most frequent mutation site, being present in 19 tumors (31.7%) in premenopausal women and 42 tumors (29.2%) in postmenopausal women. E545 was the second most frequent mutation site, and was found in 5 tumors (8.3%) among premenopausal women and 18 tumors (12.5%) among postmenopausal women. Thus, mutation frequencies detected in H1047 and E545 were similar between pre- and postmenopausal women. There were no tumors with R88Q or M1043I mutations in either pre- or postmenopausal women.

**Table 4:** *PIK3CA* mutation frequencies in breast cancer tissues in pre- and postmenopausal women

				Premenopausal	Postmenopausal
No. of patients				62	152
Wild-type				31 (50.0%)	73 (48.0%)
Mutant				29 (46.8%)	71 (46.7%)
Invalid				2 (3.2%)	8 (5.3%)
*PIK3CA* domain	Exon	Nucleotide change	Amino acid mutation	*n* = 60	*n* = 144
Single mutation				26 (43.3%)	64 (44.4%)
p85-regulatory subunit- binding	1	263 G>A	R88Q	0	0
C2	4	1035 T>A	N345K	2 (3.3%)	6 (4.2%)
	7	1258 T>C	C420R	1 (1.7%)	2 (1.4%)
Helical	9	1624 G>A	E542K	2 (3.3%)	1 (0.7%)
	9	1634 A>C^a)^, 1635 G>T^b)^, 1634 A>G^c)^, or 1633 G>A^d)^	E545X (E545A^a)^, E545D^b),^ E545G^c)^, or E545K^d)^)	4 (6.7%)	15 (10.4%)
	9	1636 C>G^e)^, 1636 C>A^f)^, 1637 A>T^g)^, or 1637 A>G^h)^	Q546X (Q546E^e)^, Q546K^f)^, Q546L^g)^, or Q546R^h)^)	0	1 (0.7%)
Kinase	20	3129 G>T	M1043I	0	0
	20	3140 A>T^i)^, 3140 A>G^j)^, or 3139 C>T^k)^	H1047X (H1047L^i)^, H1047R^j)^, or H1047Y^k)^)	17 (28.3%)	38 (26.4%)
	20	3145 G>C	G1049R	0	1 (0.7%)
Double mutations				3 (5.0%)	6 (4.2%)
C2 and kinase			N345K, H1047X	0	1 (0.7%)
Helical and kinase			E542K, H1047X	1 (1.7%)	2 (1.4%)
			E545X, G1047X	1 (1.7%)	2 (1.4%)
			E545X, G1049R	0	1 (0.7%)
			Q546X, H1047X	1 (1.7%)	0
Triple mutations				0	1 (0.7%)
Helical and kinase			E542K, Q546X, H1047X	0	1 (0.7%)

### Correlation between *PIK3CA* mutation status and clinicopathological characteristics

We next examined whether *PIK3CA* mutation status affected clinicopathological characteristics in pre- and postmenopausal women (Table 5). Invalid tumors were excluded from this analysis. In premenopausal women, patients with *PIK3CA* wild-type tumors had lower tumor grade, higher ER expression, and lower AR expression when compared to patients with *PIK3CA* mutant tumors (*P* = 0.041, *P* = 0.025, and *P* = 0.047, respectively). On the other hand, in postmenopausal women, patients with *PIK3CA* wild-type tumors had higher Ki67 LI, higher AKT phospho-Ser473, and lower ERα phospho-Ser167 when compared to patients with *PIK3CA* mutant tumors (*P* = 0.03, *P* = 0.0049, and *P* = 0.018, respectively).

**Table 5:** Correlation between *PIK3CA* mutation status and clinicopathological factors in pre- and postmenopausal women

	Premenopausal			Postmenopausal		
	Wild-type (*n* = 31)	Mutant (*n* = 29)	*P*	Wild-type (*n* = 73)	Mutant (*n* = 71)	*P*
Age (years), mean ± SD (range)	43.2 ± 5.9 (27–52)	45.4 ± 5.1 (35–56)	0.17	62.0 ± 8.2 (42–80)	63.8 ± 8.2 (47–84)	0.18
BMI, mean ± SD (range)	21.8 ± 4.1 (15.2–32.9)	21.8 ± 3.4 (16.7–32)	0.63	24.6 ± 4.4 (14–38.4)	23.8 ± 4.1 (14.2–40.9)	0.38
Tumor size (T), median (range)	1 (1–2)	1 (1–3)	0.56	1 (1–3)	1 (1–3)	0.34
No. of positive lymph nodes, median (range)	0 (0–23)	0 (0–20)	0.30	0 (0–10)	0 (0–10)	0.90
Tumor grade, median (range) mean ± SD	2 (1–3) 1.7 ± 0.6	2 (1–3) 2.0 ± 0.6	0.041^*^	2 (1–3) 2.0 ± 0.5	2 (1–3) 1.8 ± 0.6	0.051
Ki67 LI (%), median (range) mean ± SD	10.1 (0–62.7) 13.3 ± 13.6	12.2 (1.2–61.3) 15.1 ± 13.1	0.46	10.3 (2.0–52.7) 13.7 ± 10.3	7.5 (0.1–35.3) 10.2 ± 8.1	0.03^*^
ER (%), median (range) mean ± SD	90 (40–100) 83.2 ± 18.9	80 (10–100) 68.6 ± 29.1	0.025^*^	90 (10–100) 79.9 ± 24.1	90 (20–100) 83.1 ± 20.3	0.40
PgR (%), median (range) mean ± SD	90 (0–100) 65.5 ± 37.0	80 (0–100) 62.8 ± 34.7	0.44	10 (0–100) 27.7 ± 32.1	20 (0–100) 30.9 ± 31.1	0.37
AR (%), median (range) mean ± SD	1.0 (0–80) 17.4 ± 23.4	20 (1–90) 28.0 ± 26.8	0.047^*^	30 (0–95) 33.0 ± 28.9	35 (0–100) 43.6 ± 33.8	0.065
VDR (%), median (range) mean ± SD	10 (0–95) 15.4 ± 17.9	10 (0–70) 16.2 ± 17.9	0.91	10 (0–80) 15.7 ± 16.4	20 (0–50) 18.0 ± 13.1	0.13
pAKT (score), median (range) mean ± SD	120 (0–300) 146.5 ± 93.4	100 (0–230) 105.5 ± 76.9	0.14	100 (0–300) 110.2 ± 79.1	70 (0–290) 75.5 ± 65.8	0.0049^*^
pER (%), median (range) mean ± SD	20 (0–90) 25.0 ± 21.3	20 (1–95) 23.1± 21.5	0.74	15 (0–90) 17.8 ± 17.8	20 (0–90) 23.4 ± 18.0	0.018^*^

LI: labeling index; pAKT: AKT phospho-Ser473; pER: ERα phospho-Ser167.

^*^P < 0.05 is considered significant.

### Biological predictors of survival in postmenopausal women

**Figure 2:**
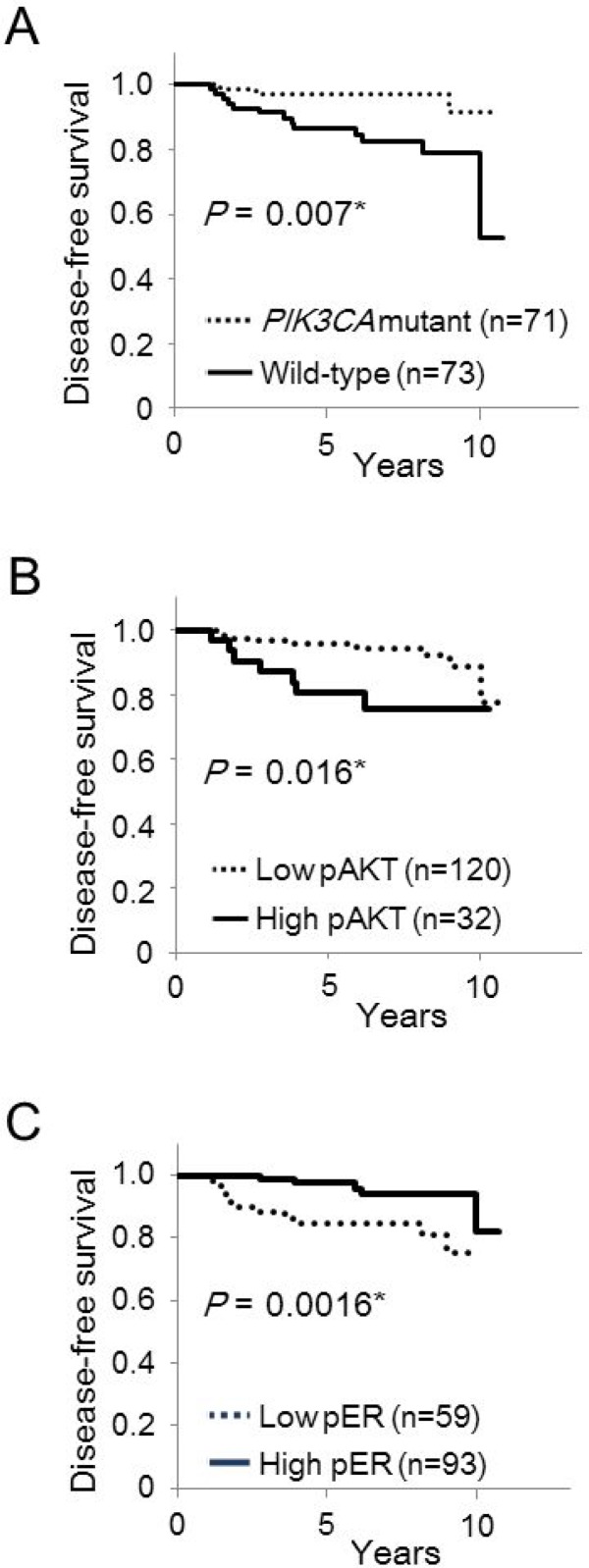
Kaplan–Meier curves of the effect of *PIK3CA* mutations, phosphorylation of AKT Ser473 and ERα Ser167 on disease-free survival in postmenopausal women Disease-free survival according to *PIK3CA* mutations (**A**), phosphorylation of AKT Ser473 (**B**), and phosphorylation of ERα Ser167 (**C**).

We then analyzed whether *PIK3CA* mutation status and phosphorylation of AKT Ser473 or ERα Ser167 affected disease-free survival in postmenopausal women (Figure 2). Postmenopausal women with *PIK3CA* wild-type tumors had significantly worse disease-free survival than patients with *PIK3CA* mutant tumors (*P* = 0.007; Figure 2A). To identify clinically meaningful cutoff points for levels of AKT phosphor-Ser473 and ERα phospho-Ser167, we performed a Kaplan–Meier analysis and verified by the log-rank test for disease-free survival. The cutoff points for the levels of AKT phospho-Ser473 and ERα phospho-Ser167 were set at 170 and 15%, respectively. Kaplan–Meier analysis showed that low AKT phospho-Ser473 was strongly associated with increased disease-free survival compared to high AKT phospho-Ser473 (*P* = 0.016; Figure 2B). Moreover, high ERα phospho-Ser167 was significantly associated with increased disease-free survival compared to low ERα phospho-Ser167 (*P* = 0.0016; Figure 2C).

We then analyzed whether clinicopathological and biological markers were associated with altered prognosis of postmenopausal patients with ER-positive, HER2-negative breast cancer (Table 6). Univariate analysis demonstrated significant association between improved disease-free survival and low AKT phospho-Ser473 (*P* = 0.02), high ERα phospho-Ser167 (*P* = 0.02) and the presence of *PIK3CA* mutations (*P* = 0.02), as well as negative lymph node status (*P* = 0.04), low tumor grade (*P* = 0.01) and low Ki67 LI (*P* < 0.001). In multivariate analysis, a significant association was observed between disease-free survival and the presence of *PIK3CA* mutations (*P* = 0.02), as well as negative lymph node status (*P* = 0.008) and low Ki67 LI (*P* = 0.005). Expression of AR and VDR was not significantly correlated with disease-free survival in the univariate analysis.

**Table 6:** Univariate and multivariate analysis of factors predicting disease-free survival in postmenopausal women

Factor	Univariate			Multivariate		
	RR^a^	95% CI^b^	*P*	RR^a^	95% CI^b^	*P*
BMI	1.02	0.91–1.15	0.68			
Tumor size	2.3	1.00–5.27	0.05			
Lymph node status	1.65	1.01–2.69	0.04^*^	1.46	1.10–1.93	0.008^*^
Tumor grade	3.3	1.31–8.34	0.01^*^	1.13	0.33–3.95	0.84
Ki67 LI	1.07	1.03–1.11	<0.001^*^	1.05	1.02–1.12	0.005^*^
ER	1.01	0.99–1.04	0.40			
PgR	0.98	0.96–1.00	0.05			
AR	1.00	0.98–1.01	0.71			
VDR	1.00	0.95–1.02	0.53			
pAKT (<170, 170≥)	3.17	1.18–8.54	0.02^*^	0.27	0.07–1.01	0.05
pER (<15%, 15%≥)	0.27	0.09–0.79	0.02^*^	2.49	0.73–8.51	0.14
*PIK3CA* mutation	0.21	0.06–0.73	0.02^*^	0.14	0.03–0.76	0.02^*^
Adjuvant chemotherapy	0.77	0.10–5.84	0.80			
Adjuvant endocrine therapy	1.5	0.48–4.66	0.48			

LI: labeling index; pAKT: AKT phospho-Ser473; pER: ERα phospho-Ser167.

^a^Relative risk.

^b^Confidence interval.

^*^P < 0.05 is considered significant.

### Combining *PIK3CA* mutation status with the status of AKT Ser473 and ERα Ser167 phosphorylation provides an improved predictor of clinicopathological characteristics and prognosis in postmenopausal women

**Figure 3:**
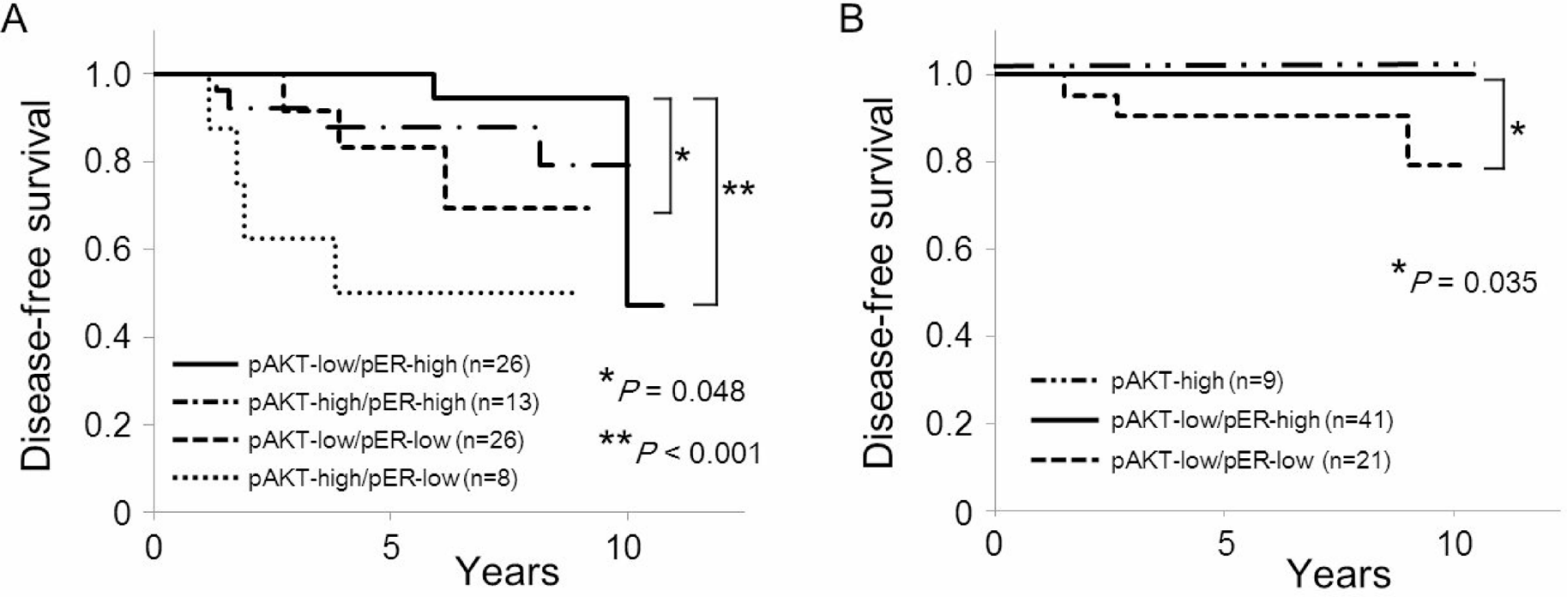
Kaplan–Meir curves of the effect of phosphorylation of AKT Ser473 and ERα Ser167 by *PIK3CA* mutation status on disease-free survival in postmenopausal women (**A**) Disease-free survival according to phosphorylation status of AKT Ser473 and ERα Ser167 in *PIK3CA* wild-type tumors. (**B**) Disease-free survival according to phosphorylation status of AKT Ser473 and ERα Ser167 in *PIK3CA* mutant tumors.

We further analyzed correlation whether a combination of *PIK3CA* mutation status, AKT phospho-Ser473 (pAKT) and ERα phospho-Ser167 (pER) had any predictive function with regard to clinicopathological characteristics and disease-free survival in postmenopausal ER-positive, HER2-negative breast cancer (Table 7). According to the *PIK3CA*-pAKT-pER status, we classified *PIK3CA* wild-type tumors into 4 types (A, B, C, and D) and *PIK3CA* mutant tumors into 3 types (E, F, and G). From a total of 73 patients with *PIK3CA* wild-type tumors, 21 patients (28%) had high AKT phospho-Ser473 (Type A or Type B), and 52 patients (72%) had low AKT phospho-Ser473 tumors (Type C or Type D). Tumor grade was significantly higher in Type A (pAKT-high/pER-low) compared with that in Type B (pAKT-high/pER-high) (*P* = 0.0083). PgR expression was significantly lower (*P* = 0.047) and VDR expression was significantly higher (*P* = 0.032) in Type B compared to those in Type C (pAKT-low/ pER-low). Four (50%) patients in Type A relapsed within 5 years of the initial treatment, whereas none of the patients in Type D (pAKT-low/pER-high) relapsed within 5 years (Table 7 and Figure 3A, *P* < 0.001). However, one patient in Type B, one patient in Type C and two patients in Type D relapsed 5 years after the initial treatment.

**Table 7:** Correlation between combined *PIK3CA*-pAKT-pER status and clinicopathological factors and prognosis in postmenopausal women

	*PIK3CA* wild-type (*n* = 73)				*PIK3CA* mutant (*n* = 71)		
Type	Type A	Type B	Type C	Type D	Type E	Type F	Type G
pAKT	high	high	low	low	high	low	low
pER	low	high	low	high	low/high	low	high
No. of patients	8 (11%)	13 (18%)	26 (36%)	26 (36%)	9 (13%)	21 (30%)	41 (58%)
Age (years), mean ± SD (range)	63.8 ± 8.0 (52–78)	59.4 ± 6.6 (50–74)	62.3 ± 9.4 (42–80)	62.4 ± 7.8 (50–77)	57.2 ± 6.8^d^ (47–71)	64.4 ± 5.9 (52–74)	65.0 ± 9.0^d^ (48–84)
BMI, mean ± SD	23.4 ± 4.2	23.2 ± 2.4	26.1 ± 4.1	24.0 ± 5.3	21.5 ± 3.0	25.3 ± 4.2	23.6 ± 4.1
Tumor size (T), median (range)	2 (1–3)	1 (1–2)	2 (1–3)	1 (1–2)	1 (1–3)	1 (1–3)^e^	1 (1–2)^e^
No. of positive lymph nodes, median (range)	1 (0–6)	0 (0–1)	0 (0–4)	0 (0–10)	0 (0–1)	0 (0–10)	0 (0–7)
Tumor grade, median (range)	3 (2–3)^a^	2 (1–2)^a^	2 (1–3)	2 (1–3)	2 (1–2)	2 (1–3)	2 (1–3)
Ki67 LI (%), median (range)	18.8 (6.8–39.8)	19.5 (3.5–52.7)	9.1 (2.0–31.7)	11.0 (2.0–31.4)	5.5 (2.0–27.6)	11.5 (0.1–34.1)	7.0 (0.4–35.3)
ER (%), median (range)	90 (20–100)	90 (10–100)	80 (10–100)	90 (20–100)	60 (50–100)	90 (20–100)^f^	90 (40–100)^f^
PgR (%), median (range)	20 (0–100)	5 (0–80)^b^	30 (0–100)^b^	10 (0–70)	5 (0–30)	20 (0–90)	20 (0–100)
AR (%), mean ± SD (range)	31.3 ± 25.7 (5–80)	42.5 ± 32.6 (1–95)	23.4 ± 21.8 (0–80)	38.3 ± 32.5 (0–95)	45.2 ± 39.6 (1–95)	29.8 ± 28.0^g^ (0–95)	50.2 ± 34.0^g^ (1–100)
VDR (%), median (range)	7.5 (0–50)	20 (0–50)^c^	5 (0–30)^c^	15 (0–80)	20 (0–50)	25 (0–40)	15 (0–50)
No. of patients with recurrence							
within 2 years	3 (38%)	0	2 (8%)	0	0	1 (5%)	0
within 5 years	4 (50%)	2 (15%)	3 (12%)	0	0	2 (10%)	0
within 10 years	4 (50%)	3 (23%)	4 (15%)	2 (8%)	0	3 (14%)	0

LI: labeling index; pAKT: AKT phospho-Ser473; pER: ERα phospho-Ser167.

^a^*P* = 0.0083 by Steel’s test.

^b^*P* = 0.047 by Steel’s test.

^c^*P* = 0.032 by Steel’s test.

^d^*P* = 0.0095 by Dunnett’s test.

^e^*P* = 0.0094 by Steel’s test.

^f^*P* = 0.023 by Steel’s test.

^g^*P* = 0.024 by Dunnett’s test.

Of the 71 patients with *PIK3CA* mutant tumors, 9 patients (13%) had high AKT phospho-Ser473 (Type E), 21 patients (30%) had low AKT phospho-Ser473 and low ERα phospho-Ser167 (Type E), and 41 patients (58%) had low AKT phospho-Ser473 and high ERα phospho-Ser167 tumors (Type G). Age was significantly lower in Type E (pAKT-high) than in Type G (pAKT-low/pER-high) (*P* = 0.0095). On the other hand, tumor size was significantly larger (*P* = 0.0094), and expression of ER and AR was significantly lower in Type F (low pAKT/ low pER) than those in Type G (*P* = 0.023 and *P* = 0.024, respectively). All of the patients with Type E or Type G are currently disease-free (Table 7 and Figure 3B). Only three patients (14%) with *PIK3CA* mutant tumors, all of whom were classified in Type F, relapsed within 10 years (Table 7 and Figure 3B, *P* = 0.035).

## DISCUSSION

*PIK3CA* mutations lead to AKT activation and induce oncogenic transformation in experimental models [20–22]. PI3K pathway activation is suggested to engender resistance to endocrine therapy in ER-positive advanced breast cancer. In support of this, progression-free survival was significantly longer in patients with ctDNA *PIK3CA* mutations who were treated with a PI3K inhibitor in combination with endocrine therapy compared to those treated with endocrine therapy alone [23]. In contrast, in early breast cancer, previous studies demonstrated that *PIK3CA* mutations were associated with a good prognosis in ER-positive, HER2-negative breast cancer [24]. Sabine and colleagues reported that mutations in *PIK3CA* were identified in almost 40% of the 4,294 postmenopausal patients with ER-positive breast cancer who participated in the TEAM trial, whereas the frequency of AKT1 mutation was 3.2% [25]. They also demonstrated that patients with *PIK3CA* mutant tumors were at a significantly lower risk of distant metastases at 5 years compared to patients without *PIK3CA* mutations. Loi and colleagues showed that in ER-positive, HER2-negative, *PIK3CA* mutant breast cancers, despite apparent PI3K/AKT pathway activation, downstream mTOR1 signaling was not greatly elevated at the transcriptional and biological levels. One of their hypotheses for underlying the mechanism is that *PIK3CA* mutations are associated with weak pathway activation, and that other PI3K pathway alterations produce stronger pathway activation [24].

Here, we showed that the levels of ERα phospho-Ser167 were significantly higher in postmenopausal ER-positive, HER2-negative breast cancers that harbored *PIK3CA* mutations when compare to their wild-type counterparts. Moreover, ERα phospho-Ser167 is associated with good prognosis. Thus, *PIK3CA* mutation seems to be associated with phosphorylation of ERα Ser167 and a response to endocrine therapy. Moreover, ERα Ser167 phosphorylation may not be due to activation of the AKT-mTOR pathway, but rather a consequence of activation of factors downstream of *PIK3CA* mutation. We suggest that *PIK3CA* is one of the key driver genes for estrogen-dependent progression in postmenopausal ER-positive early breast cancer.

On the other hand, in premenopausal women, the levels of AKT phospho-Ser473 or ERα phospho-Ser167 were not correlated with *PIK3CA* mutation status. This was, despite the presence of *PIK3CA* mutations in almost 50% of premenopausal ER-positive, HER2-negative breast cancer, a frequency identical to that in postmenopausal women. Furthermore, the levels of AKT phospho-Ser473 were significantly higher in premenopausal women compared to those in postmenopausal women, although the levels of ERα phospho-Ser167 were similar between these two groups. Thus, the downstream signaling events and clinical role of *PIK3CA* mutations in premenopausal ER-positive breast cancer require further elucidation. The mechanisms of development and biological characteristics of ER-positive breast cancer might therefore differ according to menopausal status [12, 26].

In postmenopausal breast cancer, we classified *PIK3CA* wild-type breast tumors into 4 types and *PIK3CA* mutant tumors into 3 types according to the phosphorylation status of AKT Ser473 and ERα Ser167. This revealed that a half of the patients who had *PIK3CA* wild-type tumors with high AKT phospho-Ser473 and low ERα phospho-Ser167 relapsed within 5 years after the initial treatment (Type A). The tumors of this type were of a higher grade, and none of the Type A patients relapsed 5 years after the initial treatment. Therefore, *PIK3CA* wild-type tumors with high AKT phospho-Ser473 and low ERα phospho-Ser167 might be luminal B-like tumors with relatively rapid growth and aggressive phenotype. AKT1 activation has been linked to endocrine resistance [27], and treatment of early breast cancer with mTOR inhibitors reduces AKT signaling and proliferation [28, 29]. Standard adjuvant chemotherapy including anthracyclins and/or taxanes as well as endocrine therapy might not be sufficient to block early recurrence in *PIK3CA* wild-type tumors with high AKT phospho-Ser473 and low ERα phospho-Ser167. Therefore, other strategies, such as the inclusion of inhibitors of other signal transduction pathways might improve survival for women with luminal B-like breast cancer subtype in the absence of *PIK3CA* mutation.

Our study showed that *PIK3CA* mutant tumors with low AKT phospho-Ser473 and high ERα phospho-Ser167 (Type G) was the most frequent type in postmenopausal women, and almost 30% of postmenopausal ER-positive, HER2-negative breast cancer were classified as this type. All of the patients in Type G are currently disease-free. Thus, *PIK3CA* mutant tumors with low AKT phospho-Ser473 and high ERα phospho-Ser167 appear to be of the luminal A-like subtype, and are highly endocrine-responsive. We previously demonstrated that high ERα phospho-Ser167 levels were associated with an improved disease-free and overall survival [9], and that this marker correlated with a better response to endocrine therapy and longer survival after relapse [10]. Thus, phosphorylation of ERα Ser167 might occur frequently in response to estrogen and be predictive of response to endocrine therapy in postmenopausal ER-positive breast cancer. Evaluation of ERα Ser167 phosphorylation, as well as *PIK3CA* mutation status, might be helpful in identifying patients who are likely to benefit from endocrine therapy alone versus those who are not.

In conclusion, we demonstrate that almost 50% of ER-positive, HER2-negative early invasive ductal carcinoma of the breast had mutations of *PIK3CA* gene. *PIK3CA* tumor mutations were associated with significant better disease-free survival in postmenopausal women. Furthermore, low levels of AKT phospho-Ser473 and high levels of ERα phospho-Ser167 correlated with improved survival in postmenopausal women. We therefore suggest that ERα activation, in addition to *PIK3CA* mutation, should be considered as a predictor of highly endocrine-responsive tumors and improved prognosis in postmenopausal ER-positive early breast cancer.

## MATERIALS AND METHODS

### Patients and breast cancer tissues

A total of 214 women treated for Stage I to III breast cancer between 2004 and 2010 at Hokkaido University Hospital were recruited to this study (Table 1). The study protocol was approved by the institutional review board and conformed to the guidelines of the 1996 Declaration of Helsinki. Written informed consent for the use of surgically resected tumor tissues was provided by all patients prior to treatments. The samples were chosen from a continuous series of ER-positive, HER2-negative invasive ductal carcinoma of the breast. All patients had undergone mastectomy or lumpectomy. Tumor samples were obtained during surgery. Patients received adequate endocrine or chemotherapy as adjuvant therapy. Patients treated with neoadjuvant chemotherapy were excluded. Patients received adjuvant endocrine therapy (tamoxifen ± luteinizing hormone-releasing hormone agonist for premenopausal women and aromatase inhibitors for postmenopausal women), and patients who had positive results of axillary lymph node dissection received adjuvant chemotherapy including anthracyclines and/or taxanes, as well as adjuvant endocrine therapy.

### Immunohistochemical (IHC) analysis

One 4-µm section of each submitted paraffin block was stained first with hematoxylin-eosin to verify that an adequate number of carcinoma cells were present and that the fixation quality was adequate for IHC analysis. Serial sections (4 µm) were then prepared from selected blocks and float-mounted on adhesive-coated glass slides for IHC. Details of antibodies and evaluation methods are described in Table 2 [26], and representative images of staining for AR, VDR, AKT phospho-Ser473, and ERα phospho-Ser167 are shown in Figure 1. Tumors with ≥1% of cells showing positive nuclear staining for the expression of ER were evaluated as ER-positive. To determine the level of HER2 expression, the membrane staining pattern was estimated and scored on a scale of 0 to 3+. Tumors with a score of 2+ were tested for gene amplification by FISH using the PathVysion assay (Vysis, Abbott Laboratories, Abbott Park, IL, USA). A ratio of HER2 gene/chromosome 17 > 2.2 was considered positive. Tumors were considered HER2-positive if IHC staining scored 3+ or FISH was positive [30]. HER2-positive tumors were excluded from this study. The Ki67 labeling index (LI) was assessed as the percentage of tumor cells showing definite nuclear staining among >1000 invasive tumor cells analyzed using a NanoZoomer 2.0-HT (Hamamatsu photonics, Hamamatsu, Japan) for slide scanning and Tissue Studio (Definiens, Munich, Germany) for automated scoring [31]. If there were clear hot spots, data from these areas were assessed. Phosphorylation of AKT Ser473 was quantified as the percentage of cells with positive cytoplasmic staining. An intensity score represented the average intensity of the positive cells as follows: 0 (none), 1 (weak), 2 (intermediate), and 3 (strong). The proportion of positive cells and an intensity score were then multiplied to obtain a total score, which could range from 0 to 300.

### DNA isolation and *PIK3CA* mutation analysis

Genomic DNA was extracted from formalin-fixed paraffin-embedded tumor blocks using the cobas^®^ DNA Sample Preparation Kit (Roche Molecular Systems, Pleasanton, USA) [32]. Four to eight pieces of deparaffinized 10 μm section were used for the extraction process. When tumor cells constituted less than 20% of the specimens, the tumor area was macro-dissected before DNA extraction. The amount of genomic DNA was spectrophotometrically determined (NanoDrop 2000c, Thermo Fisher Scientific, Waltham, USA) and adjusted to a fixed concentration before use for amplification/detection.

Mutations in the *PIK3CA* gene were detected using the cobas^®^ PIK3CA Mutation Test (Roche Molecular Systems) [32]. This test uses a pool of primers divided into three different mixes for each sample and control that define specific base-pair (bp) sequences that range from 85 to 155 bp in exons 1, 4, 7, 9, and 20 of the *PIK3CA* gene. An additional primer pair, targeting a conserved 167 bp region in *PIK3CA* exon 3, provided a full process control. A derivative of Thermus species Z05-AS1 DNA-polymerase is utilized for amplification. Selective amplification of target nucleic acid from the sample is achieved in the cobas^®^ PIK3CA Mutation Test by the use of AmpErase (uracil-N-glycosylase) enzyme and deoxyuridine triphosphate (dUTP). The test is designed to detect R88Q in exon 1, N345K in exon 4, C420R in exon 7, E542K, E545X (E545A, E545D, E545G, and E545K), and Q546X (Q546E, Q546K, Q546L, and Q546R) in exon 9, and M1043I, H1047X (H1047L, H1047R, and H1047Y), and G1049R in exon 20, when the mutation is present at levels of 5% or greater. All of the seventeen mutations are missense mutations. The target DNA was amplified and analyzed on the cobas^®^ z480 analyzer (cobas^®^ 4800 System, Roche). In cases of invalid results, specimen testing was repeated. An invalid result from retesting was excluded from the analysis as an invalid sample.

### Statistical analysis

Mann-Whitney *U* tests, Fisher`s exact test and the chi-square test were used to compare expression and phosphorylation of biological markers between pre- and postmenopausal women, and to compare clinicopathological and biological factors between *PIK3CA* wild-type and mutant tumors. Estimation of disease-free survival was performed using the Kaplan–Meier method, and differences between survival curves were assessed using the log-rank test. Cox’s proportional hazards model was used for univariate and multivariate analyses of prognostic values. In order to determine whether the clinicopathological and biological factors were different dependent on *PIK3CA* status, we first evaluated the homogeneity of variance using Bartlett’s test. Dunnett’s test was then used for analyzing homogenous data, and Steel’s test was used for analyzing heterogeneous data. Statistical analysis was performed using Excel software (Excel 2013 for Windows, Microsoft corp., Albuquerque, USA).

## Abbreviations

ER: estrogen receptor
Ser: serine
PgR: progesterone receptor
HER2: human epidermal growth factor receptor type 2
AR: androgen receptor
VDR: vitamin D receptor
PIK3CA: phosphatidylinositol-4,-5-bisphosphate 3-kinase catalytic subunit alpha
PI3K: phosphatidylinositol 3-kinase
IHC: immunohistochemistry
LI: labeling index
